# Impact of dumpsite compost on heavy metal accumulation in some cultivated plants

**DOI:** 10.1186/s13104-025-07083-9

**Published:** 2025-01-17

**Authors:** Basma T. Abd-Elhalim, Mathew Gideon, Kuzmin Anton, Mercy O. Boyi

**Affiliations:** 1 Department of Agricultural Microbiology, Faculty of Agriculture, Ain Shams University, Hadayek Shubra, Cairo, 11241 Egypt; 2 Department of Environment, Ministry of Environment and Natural Resources, Kaduna, Kaduna State Nigeria; 3 Department of Agricultural Products Processing, Mordovia State University, Saransk, Russia; 4 Department of Chemistry, FacultyofScience, Nigerian Defence Academy Kaduna State, Kaduna, Nigeria

**Keywords:** Dumpsite compost, Heavy metals, Human and animal health, Maize plant, Spinach plant

## Abstract

The study examines the concentrations of heavy metals in agricultural soil, compost from landfills, maize plants, and spinach crops. The results show that compost from landfills had levels exceeding EU requirements for Cd, Cu, Ni, Pb, and Zn. However, agricultural soil contained trace amounts of heavy metals. The order of metal absorption by plants was Cd > Pb > Cu > Ni > Cr > Zn > Fe > Mn. This highlights potential health risks associated with consuming crops grown in compost or soil tainted with heavy metals.

**Supplementary Information:**

The online version contains supplementary material available at 10.1186/s13104-025-07083-9.

## Background

Uncontrolled waste disposal in African countries like Egypt, South Africa, and Nigeria is causing environmental degradation due to inadequate waste management techniques. This leads to the accumulation of pollutants, particularly in soil and groundwater, which are essential storage areas. Metal contamination from open garbage disposal, industrial processes, and automobile emissions can pose health and ecosystem risks [1–16]. Dumpsite soils, rich in essential nutrients like calcium, magnesium, potassium, and salt, have been extensively studied for heavy metal contamination, revealing potential environmental deterioration and potential consequences [17–26].

Heavy metals, causing toxicity, inhibiting biodegradability, and singnificantly impact the environment and ecosystem health. Polluted from household, commercial, or municipal waste, they accumulate in plants, causing health concerns. Dumpsites contribute to soil heavy metal pollution, including As, Cd, Co, Cu, Fe, Hg, Mn, Pb, Ni, and Zn [27–29]. Heavy metals like lead (Pb) and cadmium (Cd) pose significant threats to soil health and agricultural productivity due to their toxicity and environmental persistence. Lead remains on the soil surface for extended periods, while cadmium moves through soil based on pH and organic matter. Soil pollution leads to higher metal levels, lower organic matter, nutrient retention, and ion exchange [30–32]. Heavy metal concentrations negatively impact soil biota by disrupting microbial processes and reducing microbial activity. Pollution reduces specific cation adsorption, altering soil pH and inhibiting enzymatic activity. Soil enzyme activity remains stable at 10 μg/g but increases to 50 μg/g lead to decreased activity, particularly in sandy loam soils. Heavy metals like Zn^2+^ and Cu^2+^ completely vanish urease activity [32–34]. Heavy metals pose a global environmental hazard, accumulating in plants and organisms. Variables like temperature, humidity, organic matter content, pH, and nutrient availability affect their absorption. Higher summer transpiration rates in spinach increase metal absorption, contaminating agricultural soil and endangering human health. Converting biomass into organic amendments can address this issue [35–56]. Due to the high expense of inorganic fertilizers, farmers are increasingly employing huge dumpsite composted soil as a soil supplement (Figure S1).

The study explores the absorption of trace metals by maize and spinach in soil from a large dumpsite in Kaduna Metropolis, focusing on its impact on heavy metal accumulation, health risks, and plant growth. It compares dumpsite compost with soil, assessing heavy metal levels and offering recommendations for safe agricultural practices.

## Materials and methods

### Description of study area

Sabon Tasha Railway Station is in Kaduna State, Nigeria's Chikun Local Government Area (Figure S2). The state is known for its short trees, shrubs, and grasses, and has seven neighbouring states: Kano, Bauchi, Plateau, Nasarawa, Abuja Federal Capital Territory, and Niger [38–40].

### Environmental characteristics

Kaduna State's natural landscape features diverse flora and soil composition, influenced by semi-arid to sub-humid climates and primarily loamy to sandy soil with clay areas.

### Regional context

Kaduna State's biological and socioeconomic characteristics are influenced by its borders with Zamfara, Katsina, Kano, Bauchi, Plateau, Nasarawa, Abuja, and Niger State. Its proximity to Nigeria's political hub and potential for interstate trade and cross-cultural interchange make it a significant region in the country [41].

### Samples collection and preparation

The study collected heavy metal samples from composted soil, garden area, and dumpsite using a careful sampling technique, extracting magnetic metals using a 1 kg bar magnet, and testing in small containers.

### Planting experiment

A garden plot with composted soil was planted with maize and spinach, without soil amendments or fertilizers. After 40 days, plants were harvested and carefully picked, cleaned, and labelled (Figure S3). Samples were sent to the Multi-User Laboratory, Chemistry Department, and Agricultural Microbiology Department for further analysis. The samples were dehydrated, dried, ground into a powder, and examined for heavy metals [41].

### Determination of heavy metals

Heavy metal analysis was conducted using Microwave Plasma- Atomic Emission Spectroscopy (MP-AES42000) with laboratory-quality hydrochloric and nitric acids from German supplier Riedel–de Haen. Materials were prepared by adhering to strict pre-treatment methodology, including cleaning, immersing in nitric acid, and drying. Samples were weighed, digested, and filtered. The actual concentration was calculated in mg/kg using an equation (1). The process involved heating, cooling, and adding deionized water to ensure precision and consistency [42, 43].

1 $$Actual\, Concentration =(Instrument\, Reading \times Dilution\, Factor) / Sample\, Weight$$

### The transfer factor (TF) of heavy metals from soil to plants

It is an important metric used to assess the extent to which plants absorb contaminants from their growing medium. The TF can be calculated using the following formula [45]:

2 $$Transfer\, Factor (TF) =Metal\, in\, Plant\, Concentration\, (mg/kg) / Metal\, in\, Soil\, Concentration\, (mg/kg)$$

### Statistical analysis

T-statistics were used to analyse three duplicate samples with a 95% confidence level. The findings are displayed as means ± standard deviations, with p < 0.01 denoting statistical significance.

### Quality assurance (QA) and quality control (QC) protocols

The study aimed to assess the impact of dumpsite compost on heavy metal accumulation in cultivated plants. To ensure reliability and accuracy, QA/QC protocols were implemented. Standardized methods were used to collect compost samples, soil, and plant materials, and proper labeling and storage conditions were maintained. Plant samples were dehydrated and ground into a fine powder for accurate measurement. Heavy metal analysis was conducted using Microwave Plasma-Atomic Emission Spectroscopy (MP-AES), with calibration standards prepared from certified stock solutions. Multiple replicates were analyzed to assess variability. Documentation of procedures and deviations was meticulous.

## Results

**Fig. 1:**
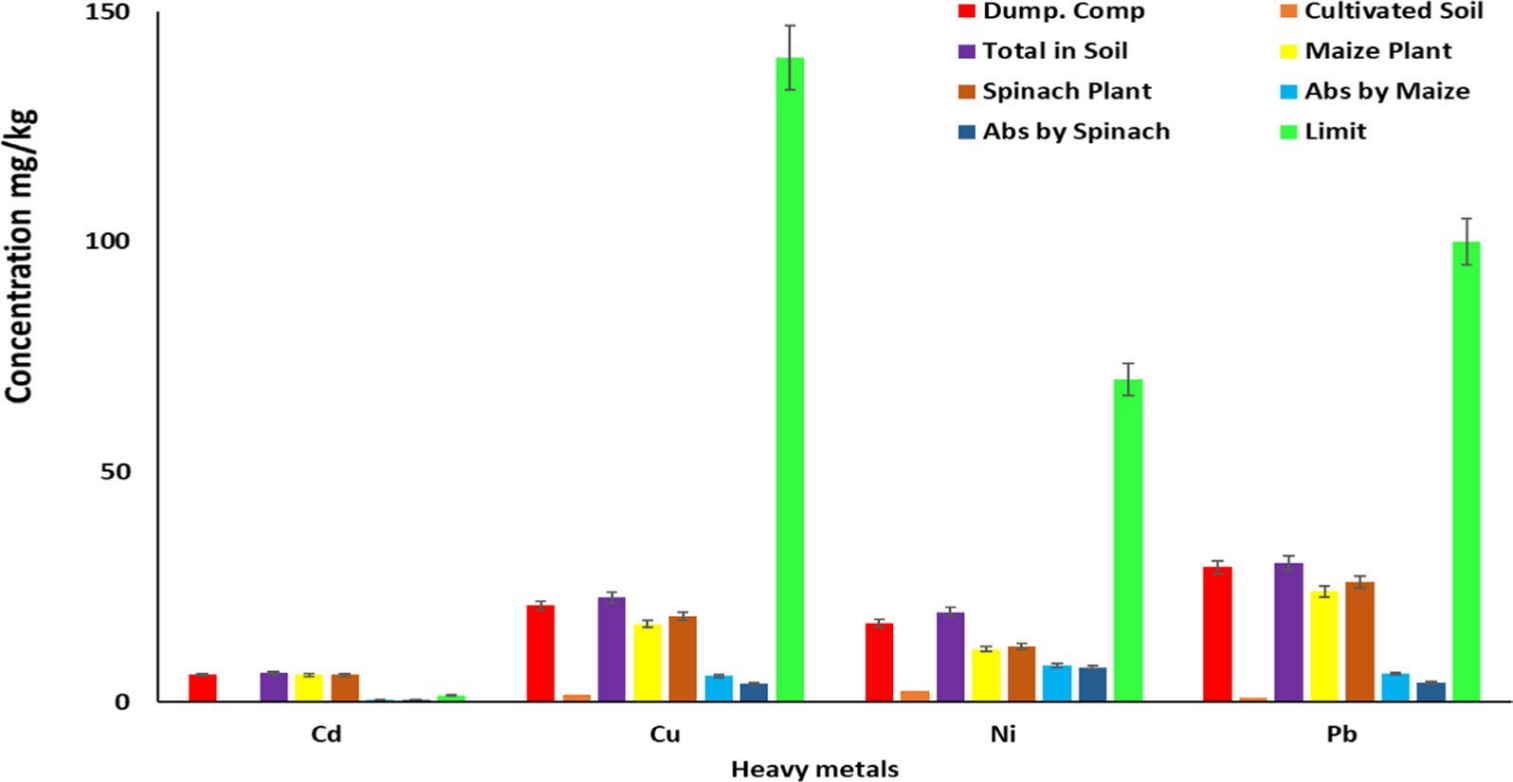
Concentration of Cd, Cu, Ni and Pb present in soil and plant samples

**Fig. 2:**
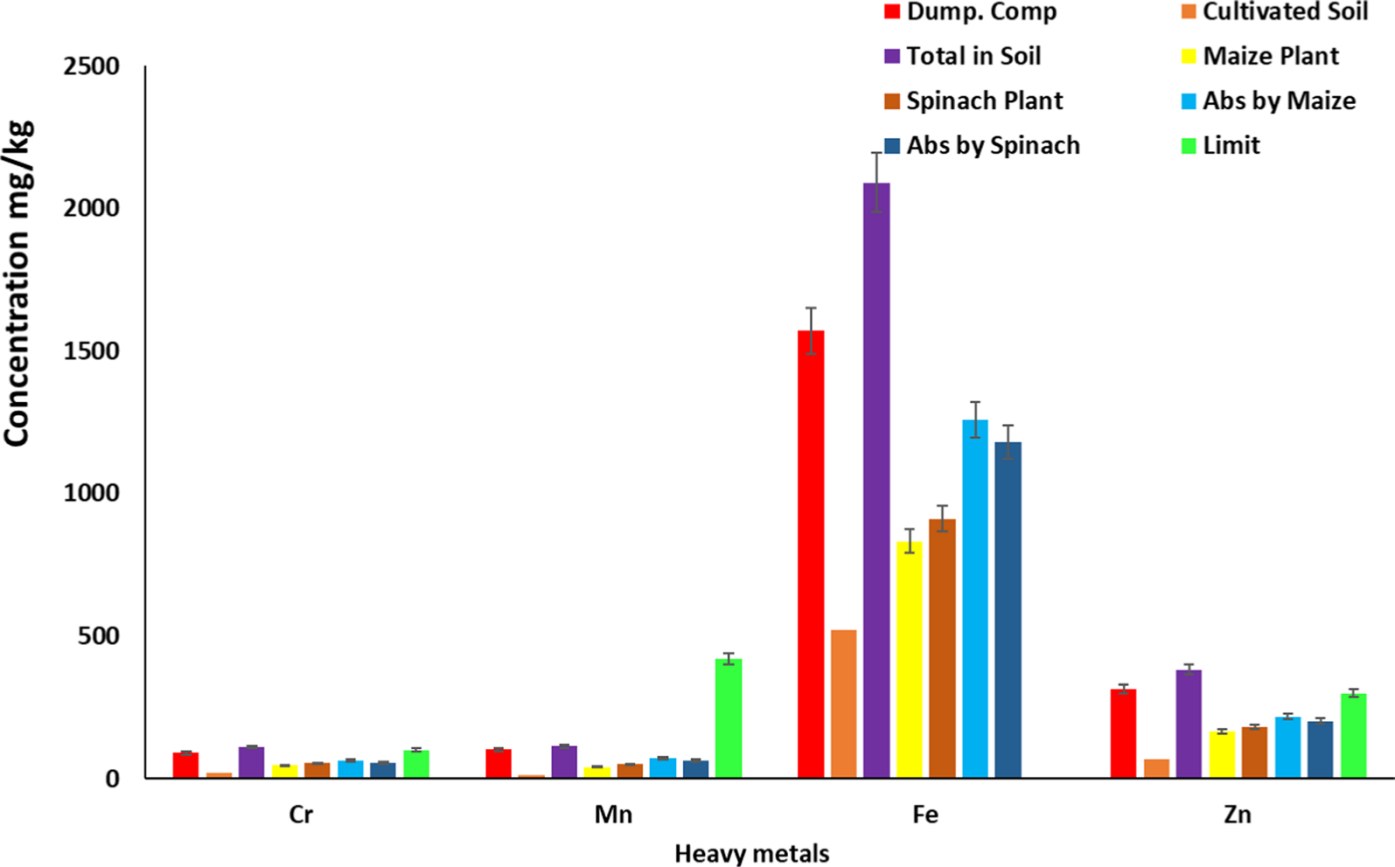
Concentration of Cr, Mn, Fe and Zn present in soil and plants samples

As shown in Table 1 and Figs. 1 and 2, the study measured heavy metal concentrations in compost from waste sites, cultivation soil, maize plants, and spinach crops, comparing them to Directive 2014/118/EU limitations. Although the concentrations were higher than EU limits, they were lower than compost from waste sites, suggesting soil mitigation. The average concentrations were less than 1.

**Table 1:** Concentrations of heavy metal of all samples analysed in mg/kg

Concentration (mg/kg)/Trace metals	Cd	Cr	Cu	Mn	Ni	Fe	Pb	Zn
Dumpsite compost	6.00 ± 0.02	89.00 ± 0.63	21.00 ± 0.32	101.00 ± 2.01	17.12 ± 0.23	1570.02 ± 15.21	29.31 ± 0.31	315.18 ± 2.23
Soil for cultivation	0.40 ± 0.01	21.31 ± 0.32	1.72 ± 0.01	12.98 ± 0.15	2.43 ± 0.08	520.11 ± 5.31	0.98 ± 0.01	67.31 ± 1.04
Total in soil	6.40 ± 0.03	110.31 ± 0.95	22.72 ± 0.33	113.98 ± 2.16	19.55 ± 0.31	2090.13 ± 20.52	30.29 ± 0.32	382.49 ± 3.27
Maize plant	5.88 ± 0.03	47.17 ± 0.51	17.01 ± 0.05	41.46 ± 0.36	11.56 ± 0.11	832.10 ± 9.57	24.07 ± 0.09	164.92 ± 3.06
Spinach vegetable	5.94 ± 0.02	54.29 ± 0.40	18.69 ± 0.17	50.53 ± 0.69	12.07 ± 0.09	910.60 ± 12.20	26.10 ± 0.12	180.45 ± 1.70
Absorbed by maize	0.52 ± 0.01	63.14 ± 0.44	5.71 ± 0.28	72.52 ± 1.8	7.99 ± 0.2	1258.03 ± 10.95	6.22 ± 0.23	217.57 ± 0.21
Absorbed by spinach	0.46 ± 0.01	56.02 ± 0.55	4.03 ± 0.16	63.45 ± 1.47	7.48 ± 0.22	1179.53 ± 8.32	4.19 ± 0.20	202.04 ± 1.57
(Directive 2014/118/EU) limit	1.50	100.00	140.00	420.00	70.00	NL	100.00	300.00

The EU does not typically set a specific limit for iron in agricultural soil as it is considered an essential nutrient

NL: No Limit

The study examines the transfer factors (TF) of maize and spinach, indicating their ability to absorb heavy metals from contaminated composted soil. The results show that both plants absorb different amounts of heavy metals, highlighting differences in their uptake capabilities. Monitoring these TFs is crucial due to potential health risks associated with consuming contaminated crops (Table 2).

**Table 2:** The calculated p-values for each heavy metal absorbed by maize and spinach

Concentration (mg/kg)/Trace metals	Cd	Cr	Cu	Mn	Ni	Fe	Pb	Zn
Total in soil	6.40 ± 0.03	110.31 ± 0.95	22.72 ± 0.33	113.98 ± 2.16	19.55 ± 0.31	2090.13 ± 20.52	30.29 ± 0.32	382.49 ± 3.27
Absorbed by maize	0.52 ± 0.01	63.14 ± 0.44	5.71 ± 0.28	72.52 ± 1.8	7.99 ± 0.2	1258.03 ± 10.95	6.22 ± 0.23	217.57 ± 0.21
TF	0.08	0.57	0.25	0.63	0.40	0.60	0.20	0.56
Absorbed by spinach	0.46 ± 0.01	56.02 ± 0.55	4.03 ± 0.16	63.45 ± 1.47	7.48 ± 0.22	1179.53 ± 8.32	4.19 ± 0.20	202.04 ± 1.57
TF	0.072	0.53	0.17	0.55	0.38	0.56	0.13	0.52
(Directive 2014/118/EU) limit	1.50	100.00	140.00	420.00	70.00	NL	100.00	300.00
p-Value	0.0030	< 0.0010	< 0.0010	0.0020	0.0380	< 0.0010	< 0.0010	< 0.0010
Significant difference	Yes	Yes	Yes	Yes	Yes	Yes	Yes	Yes

TF = Transfer factor. TF < 1: Indicates that the plant does not accumulate the metal efficiently from the soil. TF = 1: Suggests that the plant accumulates the metal at the same rate as it is available in the soil. TF > 1: Implies the plant has a high capacity to absorb the metal from the soil, which could pose health risks if these plants are consumed

## Discussion

The study examined heavy metal concentrations in soil, compost, maize, and spinach crops. Results showed significant differences in heavy metal amounts. The study used Directive 2014/118/EU limits to compare trace metal quantities. Higher levels in compost from dumpsites suggested contamination. Heavy metal levels in compost from dumpsites were within acceptable limits, while farmed soil showed lower contamination. However, cultivable soil exceeded EU Directive limits, potentially causing food safety and crop growth issues. Soil mitigation measures mitigate higher Cd, Cr, Cu, Mn, Ni, Pb, and Zn concentrations in maize plants, but still lower than compost from trash sites, potentially endangering consumer health [10, 16, 41–44]. More monitoring and remediation operations are crucial for food safety and environmental health, as the index values indicate severe pollution.

The trace metal concentration absorption index values were ranked in order of Mn, Pb, Cu, Ni, Cr, Zn, Fe, and Cd, with Cd being highly soluble in soil, particularly in acidic environments [45]. This process increases the solubility of cadmium in acidic soils with lower pH values, which increases its availability for plant absorption [46]. Additionally, compared to neutral or alkaline soils, acidic soils have greater solubility for lead (Pb), which makes it easier for plants to absorb [47]. Studies show that lead solubility increases in acidic soils, while copper solubility is moderate in acidic ones, with complex building affecting Cu availability in alkaline soils [48]. Increased solubility of nickel (Ni) in acidic conditions and the formation of soluble complexes with certain soil minerals and organic matter augment Ni's availability for plant absorption [46].

Soil pH, organic matter concentration, and redox potential affect zinc solubility, with acidic soils being more soluble due to interaction with soil minerals and organic matter [45]. Soil chromium solubility is poor, especially in trivalent states, and decreases with pH. Soil mineral and organic matter synthesis affects solubility. Iron solubility is low in aerobic conditions but increases under anaerobic conditions [47]. Research shows that manganese solubility decreases with higher pH levels, as it precipitates at higher values, affecting its availability for plant absorption [46].

The study reveals that corn absorbs more cadmium than spinach, suggesting maize may be better at absorbing it. Both crops absorb chromium differently, potentially increasing the risk of accumulation. Maize absorbs more copper than spinach, suggesting distinct bioavailability and absorption methods. Additionally, maize shows greater manganese absorption than spinach, indicating different accumulation methods.

The study reveals significant differences in the absorption of trace metals in maize and spinach. Maize absorbed nickel more than spinach, suggesting different nickel accumulation patterns. Iron absorption was also significantly different, with maize absorbing more iron than spinach. Lead absorption was significantly higher in maize, suggesting potential lead accumulation. Zinc absorption was significantly higher in maize, indicating distinct mechanisms. Understanding these differences is crucial for food safety, environmental contamination, and agricultural management strategies.

The research reveals that spinach and maize have varying capacities to absorb trace metals from polluted soil. Spinach often accumulates more metal due to its wide leaf shape, higher affinity transporters, and effective metal uptake systems, while maize may have a smaller leaf surface area and distinct root architecture [47].

A recent study found that maize grain absorbs less metal than spinach, but cattle consuming harvested plants are at risk due to soil metal content, pH, organic matter, and redox potential. Spinach may absorb more soluble metals from soil than corn [48, 49].

The study shows that organic geo-sorbents can immobilize toxic elements in mine-degraded soil, enhancing nutrient content and reducing their bioaccumulation in spinach. Specific amendments increased spinach biomass and reduced arsenic uptake, compared to control treatments [55]. Plant growth stage and chemical speciation in soil affect metal absorption dynamics. Spinach absorbs metals faster than maize due to its quick vegetative development, highlighting increased danger. Soil chemical speciation also affects metal absorption [45, 50–52].

## Conclusion

Heavy metal pollution in agricultural soil and compost from waste sites poses threats to crop development and food safety. Compost from waste sites contained high amounts of Cd, Cr, Cu, Mn, Ni, Pb, and Zn, all over EU limits. Soil intended for agriculture had lower heavy metal concentrations. Maize plants had moderate amounts of Cd, Cr, Cu, Mn, Ni, Pb, and Zn, while spinach had higher metal concentrations than maize plants. The sequence of metal absorption by plants shows considerable contamination. To reduce risks and ensure sustainable farming practices, ongoing monitoring and corrective actions are essential.

The study suggests future research on reducing heavy metal contamination in agricultural soils, using hyperaccumulator plant species, beneficial microbes, organic amendments, crop rotation, microbial bioremediation, regular monitoring, health risk assessments, and education programs, and engaging local communities in sustainable agricultural practices.

## Limitations

However, based on the context of the study, potential limitations could include:

1. Geographical scope: The study may be limited to specific regions in Egypt and Nigeria, which may not represent heavy metal accumulation in other geographical areas with different environmental conditions.
2. Sample size: The number of samples collected from compost, soil, and plants may be limited, affecting the generalizability of the results.
3. Temporal factors: Heavy metal concentrations can vary over time due to seasonal changes, agricultural practices, and waste management practices, which may not be fully captured in a single study.
4. Analytical methods: The accuracy of heavy metal measurements may depend on the sensitivity and specificity of the analytical techniques used, which could introduce variability in the results.
5. Health risk assessment: The study may not account for all potential exposure pathways or individual susceptibility factors, which could affect the overall health risk assessment.
6. Lack of longitudinal data: Without long-term monitoring, it is difficult to assess the ongoing impact of heavy metal accumulation on soil health and crop safety.

## Supplementary Information

Supplementary Material 1.

## Data Availability

All data produced or analysed during this study are included in the article.
