# Supplementary material for: Impact of dumpsite compost on heavy metal accumulation in some cultivated plants
Source: BMC Res Notes. 2025 Jan 17;18:20. doi: 10.1186/s13104-025-07083-9 (PMC11740349; doi:10.1186/s13104-025-07083-9)
Supplement: Supplementary file 1 — Supplementary Material 1. [file 13104_2025_7083_MOESM1_ESM.pdf]

# Supplementary file

## Figures legend

**Fig S1.** Excavated dumpsite compost bagged for transportation and application on farmlands. **Fig S2**. Location of study area within the state and the country.
**Fig S3.** Spinach and maize cultivated using dumpsite composted soil.

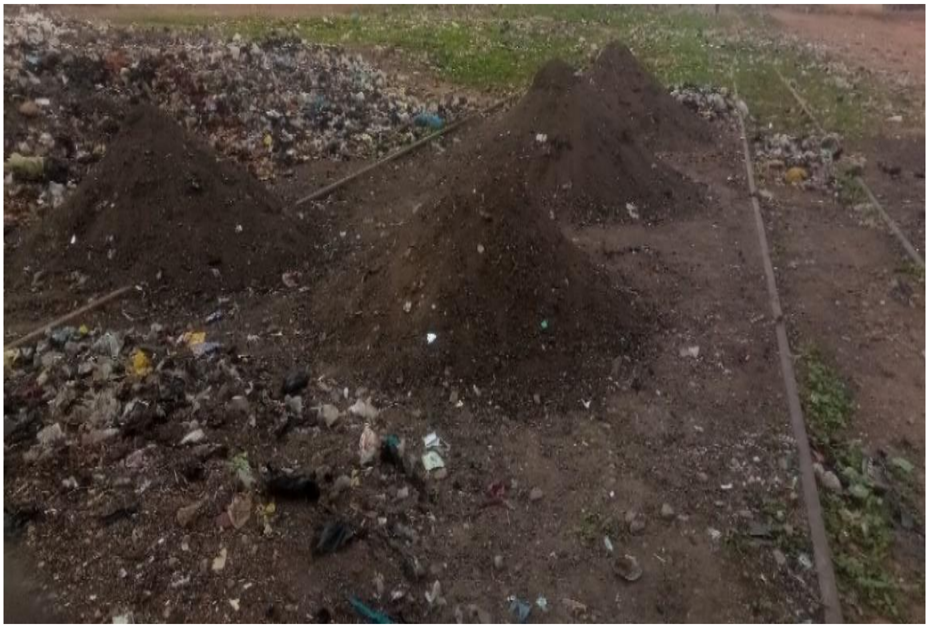

**Fig S1.**

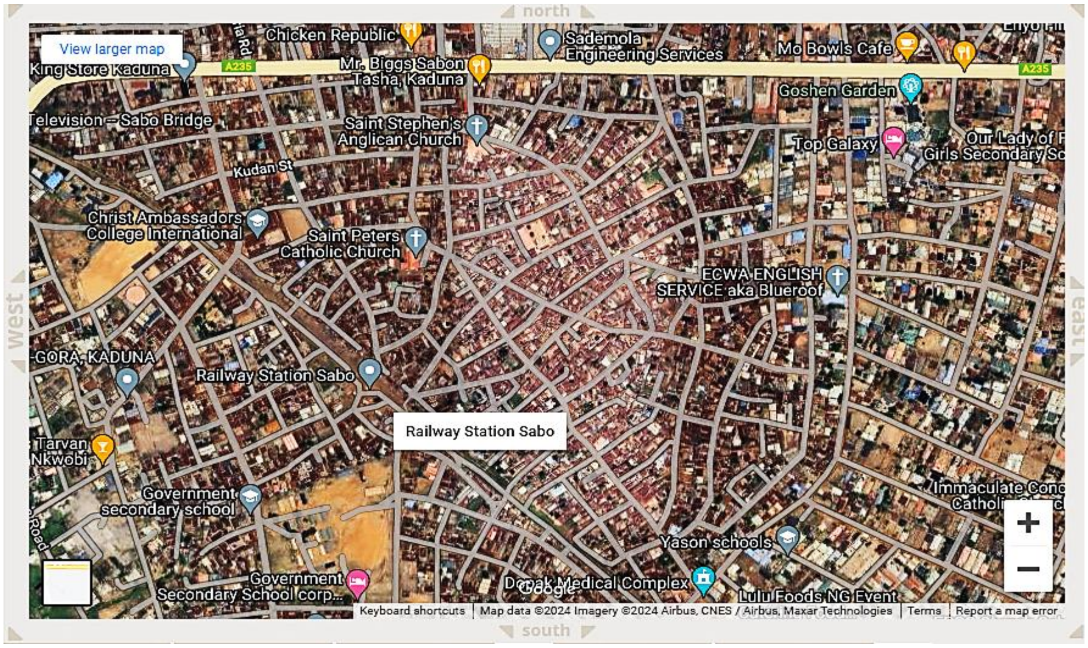


**Fig S2**.

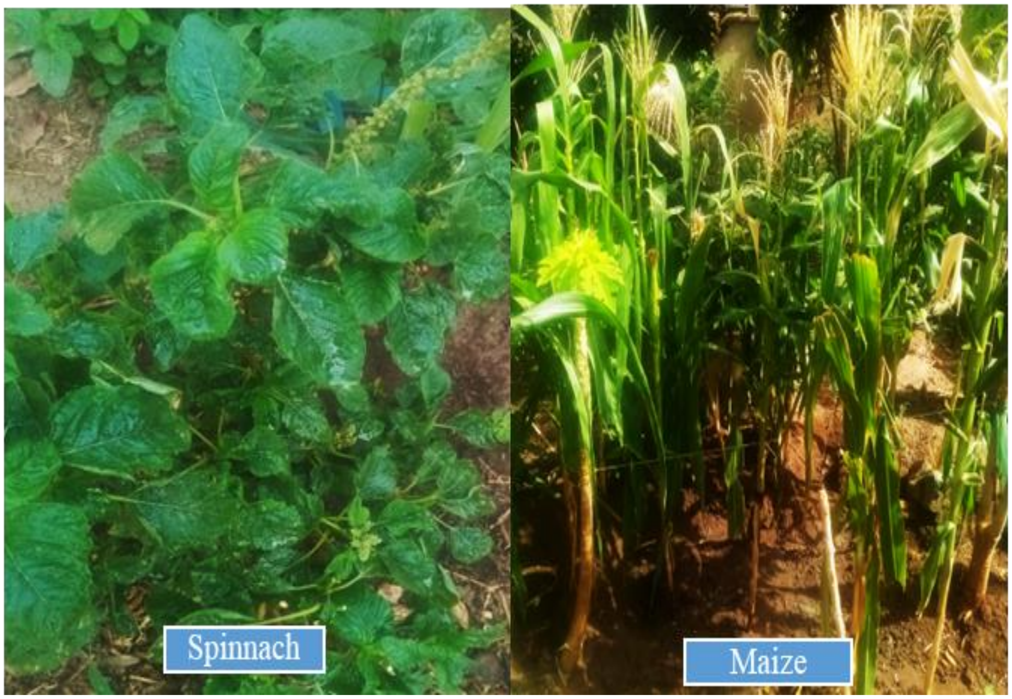


**Fig S3.**
